# Association between acute care collaborations and health care utilization as compared to stand-alone facilities in the Netherlands: a quasi-experimental study

**DOI:** 10.1097/MEJ.0000000000000969

**Published:** 2022-08-22

**Authors:** Erik M.E. Wackers, Niek W. Stadhouders, Martijn F.H. Maessen, Marit A.C. Tanke, Menno I. Gaakeer, Simone A. van Dulmen, Patrick P.T. Jeurissen

**Affiliations:** a Radboud University Medical Center, Radboud Institute for Health Sciences, IQ Healthcare, Nijmegen; b Cooperation VGZ, Arnhem; c Department of Emergency Medicine, Admiraal De Ruyter Hospital, Goes; d Ministry of Health, Welfare, and Sport, The Hague, The Netherlands

**Keywords:** after-hours care, Emergency Medical Services, health care costs, primary care

## Abstract

Health systems invest in coordination and collaboration between emergency departments (ED) and after-hours primary care providers (AHPCs) to alleviate pressure on the acute care chain. There are substantial gaps in the existing evidence, limited in sample size, follow-up care, and costs. We assess whether acute care collaborations (ACCs) are associated with decreased ED utilization, hospital admission rates, and lower costs per patient journey, compared with stand-alone facilities. The design is a quasi-experimental study using claims data. The study included 610 845 patients in the Netherlands (2017). Patient visits in ACCs were compared to stand-alone EDs and AHPCs. The number of comorbidities was similar in both groups. Multiple logistic and gamma regressions were used to determine whether patient visits to ACCs were negatively associated with ED utilization, hospital admission rates, and costs. Logistic regression analysis did not find an association between patients visiting ACCs and ED utilization compared to patients visiting stand-alone facilities [odds ratio (OR), 1.01; 95% confidence interval (CI), 1.00–1.03]. However, patients in ACCs were associated with an increase in hospital admissions (OR, 1.07; 95% CI, 1.04–1.09). ACCs were associated with higher total costs incurred during the patient journey (OR, 1.02; 95% CI, 1.01–1.03). Collaboration between EDs and AHPCs was not associated with ED utilization, but was associated with increased hospital admission rates, and higher costs. These collaborations do not seem to improve health systems’ financial sustainability.

## Introduction

Many health systems struggle with increasing numbers of emergency department (ED) visits, long wait times, and crowding. Those trends pose a long-term threat to the (efficiency of) the acute care system [1–4]. Strategies to reduce ED crowding range from input interventions, such as after-hours primary care (AHPC), throughput interventions that focus on efficient triage and treatment, and output interventions that target timely transfer to inpatient beds [5]. The effectiveness of interventions to reduce ED crowding in different hospital contexts remains contested [4,6]. Multiple health systems, such as those in the UK, Denmark, France, the Netherlands, and the USA, have invested in the concentration of urgent primary care to alleviate pressure on the ED [1,7]. The goals are to reduce unnecessary ED visits, increase patient satisfaction, and lower costs while patient outcomes remain equal [2,8].

In the Netherlands, a well-established primary sector exists where the concentration of urgent primary care has soared over the past decade [2]. Currently, many EDs collaborate with primary care physician cooperatives to provide after-hours urgent care. Self-referral rates are relatively low in the Netherlands (15% of ED visits), and hospital admission rates of patients seen in the ED are relatively high (38% of ED visits), compared with other European health systems [9]. Two million Dutch patients visit the ED annually (2012–2016), which translates to a slightly downward trend, given population growth (124 ED visits per 1000 inhabitants in 2012 and 115 ED visits per 1000 inhabitants in 2015) [9,10]. The number of ED physicians and nurses increased in the same period. Finally, the number of EDs declined by 20% [10,11], which led to a larger patient burden on the remaining EDs.

Payers see collaboration as an important lever to reduce the costs of (unnecessary) acute care treatment. Systematic reviews illustrate the lack of rigorous evaluations of the association between acute care collaboration (ACC) and ED use [12–15]. Small-scale experiments with urgent care centers (UCCs) in the UK and the USA show some tentative positive effects [16,17]. Earlier research in the Netherlands also indicated that ACCs could lead to a reduction in ED utilization and a smaller share of low-acuity patients at the ED [18,19]. However, another study showed mean episode costs to be higher in ACCs versus stand-alone acute care facilities [20]. Thus, current research has produced mixed results. Moreover, some studies were limited by a small scope [16–19]; others lacked information about follow-up treatment and costs [20]. This study adds to existent research by exploring the entire acute care chain using a large set of claims data that included information on follow-up care.

We investigated differences between patient journeys through ACCs and journeys using stand-alone acute care facilities. In this study, stand-alone acute care facilities are hospital-based EDs and AHPC providers that are not (geographically) colocated. Comparison of all ACCs and stand-alone acute care facilities may provide additional insights about volumes and total costs of the patient journey. Our primary research question: How do ACCs relate to stand-alone facilities in terms of ED utilization, hospital admissions, and total costs? It was hypothesized that collaboration between EDs and AHPC providers results in the reduction of ED visits and hospital admissions, and, subsequently, lower total costs per patient journey. A description of the Dutch acute care system can be found in Supplementary Material, Supplemental Digital Content 1, http://links.lww.com/EJEM/A350.

## Methods

In this quasi-experimental study, patients who visited ACCs were compared to those who visited stand-alone facilities between 1 January 2017 and 31 December 2017. Patient journeys were analyzed in terms of ED utilization, hospital admissions, and total patient journey costs using generalized linear models (GLMs).

This analysis was performed using claims data of one of the largest Dutch insurers, with a national market share of approximately 25% and covering 4 million insured persons [21]. Organizational data on collaboration of EDs and AHPC providers were retrieved through publicly accessible information provided by the National Institute for Public Health and the Environment [22]. Approval was obtained from the Medical Research Ethics Committee Arnhem-Nijmegen, the Netherlands (Ref. 2019-5641).

### Study population

All visits to EDs and AHPC providers in the period of 1 January 2017 to 31 December 2017 were included. Patients visiting both facilities on the same day were assumed to be referred from the AHPC provider to the ED. All patients referred or self-referred to the ED or who had registered ED activities were selected. Supplementary Material, Supplemental Digital Content 2, http://links.lww.com/EJEM/A351, demonstrates that our sample population was representative of the broader Dutch population.

Two patient journeys were distinguished: (a) patients visiting stand-alone facilities and (b) patients visiting ACCs. ACCs are characterized by shared triage, where patients are either sent to the ED or to an AHPC provider. Patients labeled to the ACC group visited a collaborating AHPC provider or a collaborating ED (or both). Patients who visited a stand-alone AHPC provider, but subsequently visited a collaborating ED, were also included in the ACC group, as they were triaged at the ACC. Patients in the stand-alone group visited a stand-alone AHPC provider or a stand-alone ED (or both). Patients who visited an ACC, but subsequently visited a stand-alone ED, were included in the stand-alone group.

During the study period, 722 183 patients visited the ED, an AHPC provider, or both. After removing from the data visits to private clinics or specialized hospitals (which do not have EDs), 720 526 patients remained. Patients visiting an ED with limited service hours, an AHPC provider open for a limited number of out-of-office hours, or an ED that could not be assigned to a correct grouping (ACC or stand-alone), were also excluded. After these exclusions, 610 846 patients remained. Ultimately, one additional record was also removed because it was incomplete, leaving a total of 610 845 unique patients for our analysis.

### Outcomes and data analysis

Patients were compared across several variables, including age, sex, number of comorbidities, and, if applicable, type of hospital and number of visits. Comorbidities were included to adjust for preexisting chronic conditions that may increase the risk of visiting an AHPC provider or ED. These conditions were derived from pharmaceutical data from 2016 to 2017, following a Swiss study on claims data used to identify chronic conditions [23].

ED utilization, hospital admissions, and discharges from AHPC providers were determined from the claims data. Incurred costs were defined as insurer claims and included (a) AHPC consultations and (b) claims for ED and follow-up care in the hospital. Claims for ED care are considered hospital expenses in the Dutch healthcare system. Both inpatient and outpatient care is financed on a diagnosis-related group base. Claims costs, therefore, also include follow-up care in the hospital. All ED visits were analyzed, as claims data do not differentiate in after-hours visits and regular visits. Self-referrals were defined as patients visiting the ED without any medical referral or ambulance transport. Descriptive statistics were used to assess baseline differences.

Logistic regressions (GLM binomial family with logit link) were used to test the association between organization (stand-alone vs. ACC) and selected dependent variables: ED utilization, hospital admissions, and discharge after AHPC visit. Age, sex, and the number of comorbidities were included as confounders. Hospital type was also included in our model to assess the association with hospital admission. Additionally, gamma regression (GLM gamma family with log link) was used to evaluate the associations between the two patient journeys and total costs (both AHPC and ED visits including follow-up care). This statistical method allowed us to adjust for a right-skewed cost distribution, which is common for health care costs. Both patient and provider characteristics were included as confounders. Furthermore, the number of visits per patient as a confounder were included, as patients with multiple visits were expected to incur higher costs. To include those patients who only visited AHPC, AHPC providers were added to the model as a separate organization type. This allowed for analysis of the total costs for the patient journey, as opposed to only costs incurred in the ED and subsequent hospital admission. Data were analyzed using RStudio (version 1.0.143; RStudio, PBC., Boston, Massachusetts, USA).

## Results

**Fig. 1:**
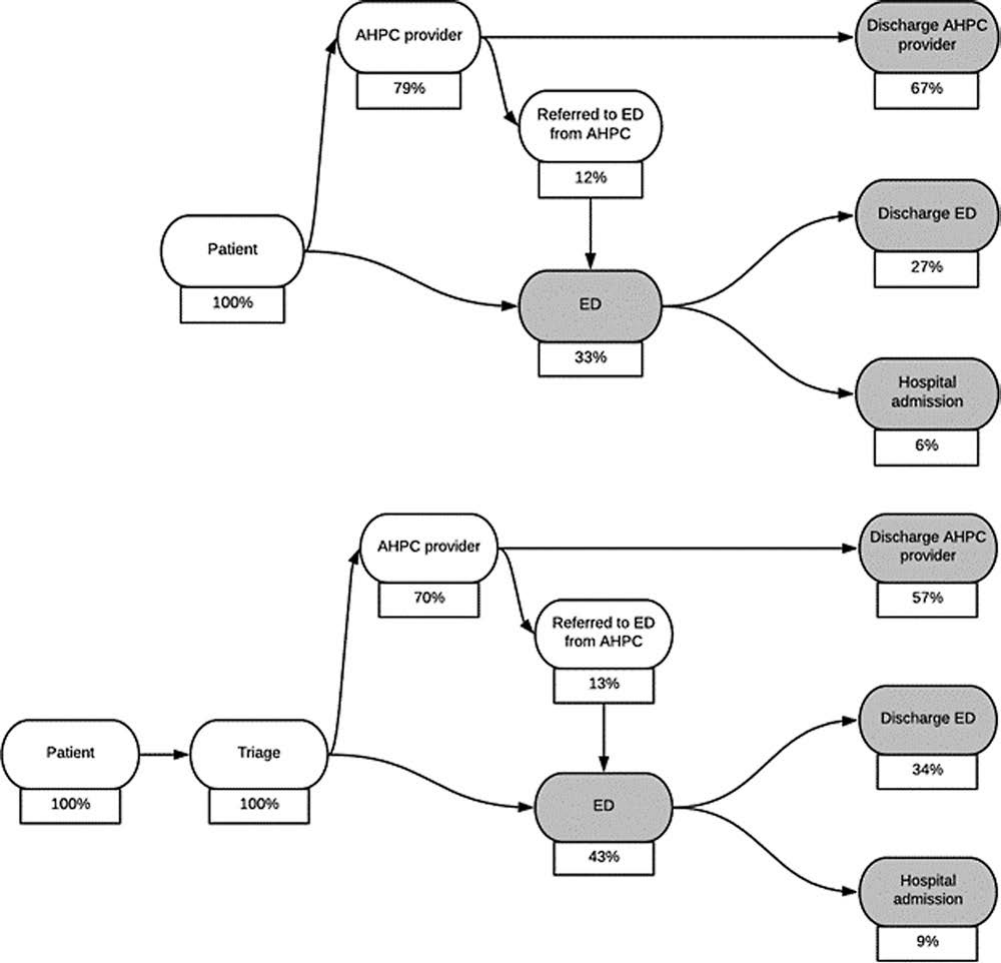
Directed acyclic graphs (DAG) of the patient journey in stand-alone acute care settings (top journey) and acute care collaborations (bottom journey); percentages based on actual data. AHPC, after-hours primary care; ED, emergency department.

Of the patients who met our inclusion criteria, 144 430 (23.6%) visited stand-alone facilities; 466 415 (76.4%) visited ACCs. Figure 1 shows full patient journeys in directed acyclic graphs. The shaded nodes of Fig. 1 were modeled using regression.

Table 1 presents descriptive data related to patient journeys and total costs. Patients in ACCs incurred higher costs in the full patient journey, including hospital follow-up care, than patients in stand-alone facilities (median costs €194 vs. €139, unadjusted). Data include patients who only visited an AHPC provider. Median costs for self-referrals in the ED were lower for patients in stand-alone facilities than ACCs (€373 vs. €421, unadjusted). The number of comorbidities is similar in both groups (1.74 on average).

**Table 1:** Baseline comparison of stand-alone facilities and acute care collaborations

Variable	Total (*N* = 610 845)	Stand-alone (*N* = 144 430–23.6%)	ACC (*N* = 466 415–76.4%)
Organizational characteristics			
Hospitals per category	77 hospitals^a^:	7 hospitals:	70 hospitals:
	42 general hospitals	2 general hospitals	40 general hospitals
	27 tertiary hospitals	2 tertiary hospitals	25 tertiary hospitals
	8 academic medical centers	3 academic medical centers	5 academic medical centers
AHPC providers	56 AHPC providers^a^	11 AHPC providers	45 AHPC providers
Most common fields at ED	37% general surgery	35.3% general surgery	37.5% general surgery
	14.6% cardiology	14.7% cardiology	14.5% cardiology
	11% internal medicine	11.2% neurology	11.1% internal medicine
	10.3% neurology	10.5% internal medicine	10.1% neurology
	7.2% orthopedics	7.6% orthopedics	7.1% orthopedics
	19.9% other	20.7% other	19.7% other
Self-referrals	12.3% of ED patients	14.3% of ED patients	11.8% of ED patients
Patient characteristics			
Sex	286 165 male (46.8%)	67 105 male (46.5%)	219 060 male (47%)
	324 680 female (53.2%)	77 325 female (53.5%)	247 355 female (53%)
Age (SD)	42.4 (26.92)	41.6 (26.96)	42.6 (26.91)
Number of comorbidities (SD)	1.74 (1.71)	1.74 (1.73)	1.74 (1.70)
Costs patient journey in €			
Total costs			
Mean (SD)	875.36 (1913.01)	780.58 (1895.24)	904.71 (1917.48)
Median (IQR)	174.82 (501.2)	139.11 (439.04)	194.47 (519.45)
Median costs self-referrals (IQR)	407.820 (492.76)	373.05 (435.54)	420.65 (526.85)

ACC, acute care collaboration; AHPC, after-hours primary care provider; ED, emergency department; IQR, interquartile range.

a Some hospitals and AHPC providers have multiple locations.

Table 2 describes the association between the type of patient journey (ACC or stand-alone) and ED utilization. Adjusted for sex, age, and number of comorbidities, no association was found between ACCs and ED utilization compared to stand-alone facilities [odds ratio (OR), 1.01; 95% confidence interval (CI), 1.00–1.03]. Second, the odds of hospital admission in ACC patient journeys compared with patient journeys in stand-alone settings were modeled. ACCs were associated with an increase of hospital admissions (OR, 1.07; 95% CI, 1.04–1.09), adjusted for age, sex, number of comorbidities, and hospital type. In comparison to academic medical centers, patients in tertiary and general hospitals were associated with an increase of inpatient stays. Third, the odds of immediate discharge after an AHPC provider visit were modeled. ACCs were associated with decreased numbers of AHPC provider discharge visits compared to stand-alone facilities (OR, 0.56; 95% CI, 0.55–0.57). These results correspond to the unadjusted data in Fig. 1.

**Table 2:** Logistic regression (generalized linear model logit link) results evaluating the odds of emergency department utilization, hospital admission, and after-hours primary care provider discharge

Variable	ED utilization (*N* = 552.048)	Hospital admission (*N* = 273.163)	AHPC provider discharge (*N* = 610.845)
Patient journey			
Stand-alone	1 (Reference)	1 (Reference)	1 [Reference]
ACC (CI)	1.01 (1.00–1.03)	1.07 (1.04–1.09)	0.56 (0.55–0.57)
Sex			
Male	1 (Reference)	1 (Reference)	1 (Reference)
Female (CI)	1.10 (1.08–1.12)	0.82 (0.80–0.83)	1.23 (1.22–1.24)
Age (years) (CI)	1.00 (1.00–1.00)	1.02 (1.02–1.02)	0.99 (0.99–0.99)
Number of comorbidities(CI)	0.92 (0.92–0.92)	1.07 (1.07–1.08)	1.00 (1.00–1.01)
Provider type			
Academic		1 (Reference)	
General (CI)		1.35 (1.29–1.41)	
Tertiary (CI)		1.32 (1.26–1.38)	

Data are presented as odds ratio (OR; 95% CI) unless otherwise noted.

ACC, acute care collaboration; AHPC, after-hours primary care provider; CI, confidence interval; ED, emergency department; OR, odds ratio.

Finally, gamma regression was used to associate the total costs of the patient journey, including hospital follow-up care, with the type of patient journey (Table 3). ACCs were associated with higher total costs for the patient journey compared to stand-alone facilities (OR, 1.02; 95% CI, 1.01–1.03). As expected, costs were higher for patients with more AHPC visits during the year and for patients visiting academic medical centers.

**Table 3:** Gamma regression (generalized linear model with log link) results evaluating total costs incurred in the patient journey (stand-alone or acute care collaboration), adjusted for sex, age, number of comorbidities, number of visits, and organization type

Variable	Total costs incurred (*N* = 610.845)
Patient journey	
Stand-alone	1 (Reference)
ACC (CI)	1.02 (1.01–1.03)
Sex	
Male	1 (Reference)
Female (CI)	0.92 (0.91–0.92)
Age (years) (CI)	1.01 (1.01–1.01)
Number of comorbidities (CI)	1.04 (1.03–1.04)
Number of visits	
AHPC provider (CI)	1.20 (1.20–1.21)
ED (CI)	1.00 (0.99–1.00)
Provider type	
Academic	1 (Reference)
General (CI)	0.78 (0.76–0.79)
Tertiary (CI)	0.84 (0.82–0.85)
Only AHPC (CI)	0.04 (0.04–0.04)

Data are presented as odds ratio (OR; 95% CI) unless otherwise noted.

ACC, acute care collaboration; AHPC, after-hours primary care provider; CI, confidence interval; ED, emergency department; OR, odds ratio.

## Discussion

Increased coordination and collaboration between EDs and AHPC providers should alleviate pressure on the EDs and increase efficiency and reduce costs. However, this study did not observe that fewer patients visited the ED or fewer patients were admitted to the hospital. We show that patients visiting ACCs were not associated with ED utilization, but were associated with an increase in hospital admissions and total reimbursement costs. Self-referrals in ACCs generally incurred higher costs compared to self-referrals in stand-alone facilities. This may indicate that self-referrals with a lower disease burden and lower costs are more often seen in a stand-alone ED. Total patient journey costs revealed higher costs for the total patient journey in ACCs.

In line with our results, Roland and Abel [24] have advised caution targeting interventions that seek to reduce emergency hospital admissions. Supplier-induced demand effects can easily occur when more services are made available [14]. Patients visiting an AHPC provider collaborating with an ED may be an example of such effects: physicians have a lower threshold for ED referrals since they are colocated. This may contribute to defensive medicine, in which available tests, procedures, and referrals are used to reduce malpractice liability [25]. Finally, self-referrals, more common in stand-alone EDs, may have a higher threshold for hospital admission than patients referred by general practitioners, who are common in ACCs [18]. Although a reduction of hospital admissions may lead to the reduction of unnecessary care in some cases, it may also lead to undertreatment in other cases. Therefore, it is recommended to analyze and compare patient outcomes in stand-alone facilities and ACCs to determine potential under- or overtreatment as well as cost-effectiveness (incremental cost-effectiveness ratio).

Colocation of primary and emergency care is contested on the international level as well, illustrated by a lack of strong evidence and mixed results [14,15]. Previous research on ED crowding showed that specific local causes are associated with the effectiveness of interventions [6]. In the Dutch setting, EDs in urban areas treat more low-acuity patients compared to other regions [11]. Therefore, collaboration between the ED and primary care providers may be more effective in urban areas. In the USA, for example, the distance patients must travel to health care providers may play a significant role in the effectiveness of UCCs [17].

Additional research on variation among acute care organizations and their respective patient populations may be useful for implementing bespoke interventions. A distinctive characteristic of acute care is that it functions as a chain economy: costs incurred in AHPC provider settings or EDs have a rippling effect on costs further along the patient journey [26]. Policymakers should evaluate the implications of interventions in this area by examining the entire chain [5].

### Limitations

Selection effects may occur since self-referrals are more common in stand-alone EDs, and those patients typically have a lower disease burden. However, this bias was limited by also including patients visiting only AHPC providers who then got dismissed. Therefore, low-acuity patients, whether self-referred to the ED or treated by an AHPC provider, were represented in both groups. Moreover, analysis of comorbidities in both groups shows no differences in preexisting conditions. Second, although the actual number of visits and the total patient costs are recorded, visits to different locations within the same AHPC group may have occurred. Therefore, some smaller inaccuracies may have resulted in linking patients to ACCs or stand-alone facilities. Our assumption is that the overwhelming majority of patients with multiple claims visited the same provider in these separate episodes. Third, data on AHPC providers were aggregated at the organizational level, somewhat blurring differences between individual locations. Therefore, some locations within the same AHPC group were part of an ACC, while others were stand-alone. If at least one location was part of an ACC, we attributed the entire group to the ACC patient journey. This approach was considered conservative, as only AHPC providers were attributed to the stand-alone group if all locations were stand-alone. Finally, reimbursement claims vary between hospitals and also deviate from actual provider costs. It is not possible to isolate actual costs for hospital EDs. Similarly, we were unable to isolate ED costs during after-hours. This limitation affects both groups of analysis, which limits potential bias.

### Conclusion

EDs and AHPC providers increasingly collaborate in an attempt to alleviate pressure on the ED and provide an efficient alternative for after-hours urgent care in the Netherlands. This study does not support this model as a valid strategy for containing costs or increasing efficiency. An increase in hospitalization and reimbursement costs as compared with stand-alone facilities has been demonstrated in this large study on retrospective claims data.

## Acknowledgements

The authors would like to thank VGZ for providing the data.

This work was supported by the Ministry of Health, Welfare and Sports in the Netherlands.

The work is attributed to Radboud University Medical Center, Radboud Institute for Health Sciences, IQ healthcare, Nijmegen, The Netherlands.

### Conflicts of interest

There are no conflicts of interest.

## Supplementary Material
