# Supplementary material for: Association between acute care collaborations and health care utilization as compared to stand-alone facilities in the Netherlands: a quasi-experimental study
Source: Eur J Emerg Med. 2022 Aug 22;30(1):15–20. doi: 10.1097/MEJ.0000000000000969 (PMC9770117; doi:10.1097/MEJ.0000000000000969)
Supplement: Supplementary file 1 [file ejem-30-15-s001.pdf]

**Supplemental Digital Content 1**

**Institutional background acute care in the Netherlands**

Patients visit the emergency department (ED) 1) upon referral by their general practitioner, 2) upon referral by an after-hours primary care (AHPC) provider 3) or self-referral In the Netherlands, general practitioners are responsible for after-hours primary care (evenings, nights, and weekends), and collaborate with AHPC providers to alleviate their workloads[1]. As in other countries, co-location of AHPC providers and EDs is rising, in order to prevent ED self-referrals. Acute care patients cannot be admitted directly into the hospital, following an AHPC provider visit. They are referred to the ED.

In this study, ACCs are defined by a geographic co-location of an ED and an AHPC provider, opposed to stand-alone facilities, in which independent EDs or AHPC providers are based in different locations. In practice, collaboration ranges from the ED and AHPC providers with separate entrances, to full integration with a shared reception desk and a common triage procedure[2]. Patients in the Netherlands may have to pay for ED visits until their yearly deductibles are met (between €385 and €885), whereas visits at AHPC providers are free[3]. The obligatory deductible is set at €385, and insured persons may voluntarily raise their deductible up to €885. An increased deductible translates to lower premiums.

AHPC providers are financed with a fixed annual budget. This budget is determined by the number of inhabitants in the service area multiplied by the negotiated capitated rate (with a maximum set by the health care authority). In sparsely populated areas, additional reimbursement up to 110% of the maximum may be granted[4].

Hospital care, including care in the ED, is financed on a diagnosis-related group (DRG) base. These are case-based payments, which include both outpatient and inpatient hospital care. The actual rates are negotiated with health insurers (70%) or set by the health care authority (30%). Emergency care does not qualify as a separate DRG. It is included with other medical treatments in a DRG. In this study, follow-up includes a hospital admission subsequent to an ED visit.

Providers for whom the ED does not generate sufficient revenue to cover their nationally set fixed costs may request financial support to cover deficits, provided closure of the ED would, however, jeopardize the 45-minute (the national guideline maximum time for the total journey of the ambulance reaching the patient and subsequently the ED)[5]. For reference, in 2017 ten EDs were categorized as “sensitive”, meaning closure would endanger accessibility of acute care in the region[6].

## References


1. Smits, M., et al., *The development and performance of after-hours primary care in the Netherlands: a narrative review.* Ann Intern Med, 2017. **166**: p. 737-742.
2. Gaakeer, M., et al., *The emergency department landscape in The Netherlands: an exploration of characteristics and hypothesized relationships.* Int J Emerg Med, 2018. **11**(1): p. 35.
3. Rijksoverheid [Dutch Government]. *Wanneer betaal ik een eigen risico voor mijn zorg? [When am I required to pay a deductible for health care?]*. n.d. 12-05-20]; Available from: https://www.rijksoverheid.nl/onderwerpen/zorgverzekering/vraag-en-antwoord/eigen-risico-zorgverzekering.
4. Nederlandse Zorgautoriteit [Dutch Healthcare Authority], *Beleidsregel huisartsendienstenstructuur [Policy Rule After-Hours Primary Care]*, in *BRREG-19138*, Nederlandse Zorgautoriteit, Editor. 2018: Utrecht.
5. Nederlandse Zorgautoriteit [Dutch Healthcare Authority], *Beleidsregel Beschikbaarheidbijdrage op aanvraag [Policy Rule Financial Support Emergency Departments]*, in *BRREG-20144*, Nederlandse Zorgautoriteit, Editor. 2019: Utrecht.
6. Rijksinstituut voor Volksgezondheid en Milieu [National Institute for Public Health and the Environment], *Aanbod en bereikbaarheid van de spoedeisende ziekenhuiszorg in Nederland 2017*. 2017: Bilthoven.
